# Supplementary material for: The interactome of intact mitochondria by cross-linking mass spectrometry provides evidence for coexisting respiratory supercomplexes
Source: Mol Cell Proteomics. 2017 Dec 8;17(2):216–32. doi: 10.1074/mcp.RA117.000470 (PMC5795388; doi:10.1074/mcp.RA117.000470)
Supplement: Supplemental Data [file supp_RA117.000470_133922_0_supp_23978_fzffwf.zip › spectra_annotation/mito_DR_spectra_annotation/284-1-1-1-8-1.pdf]

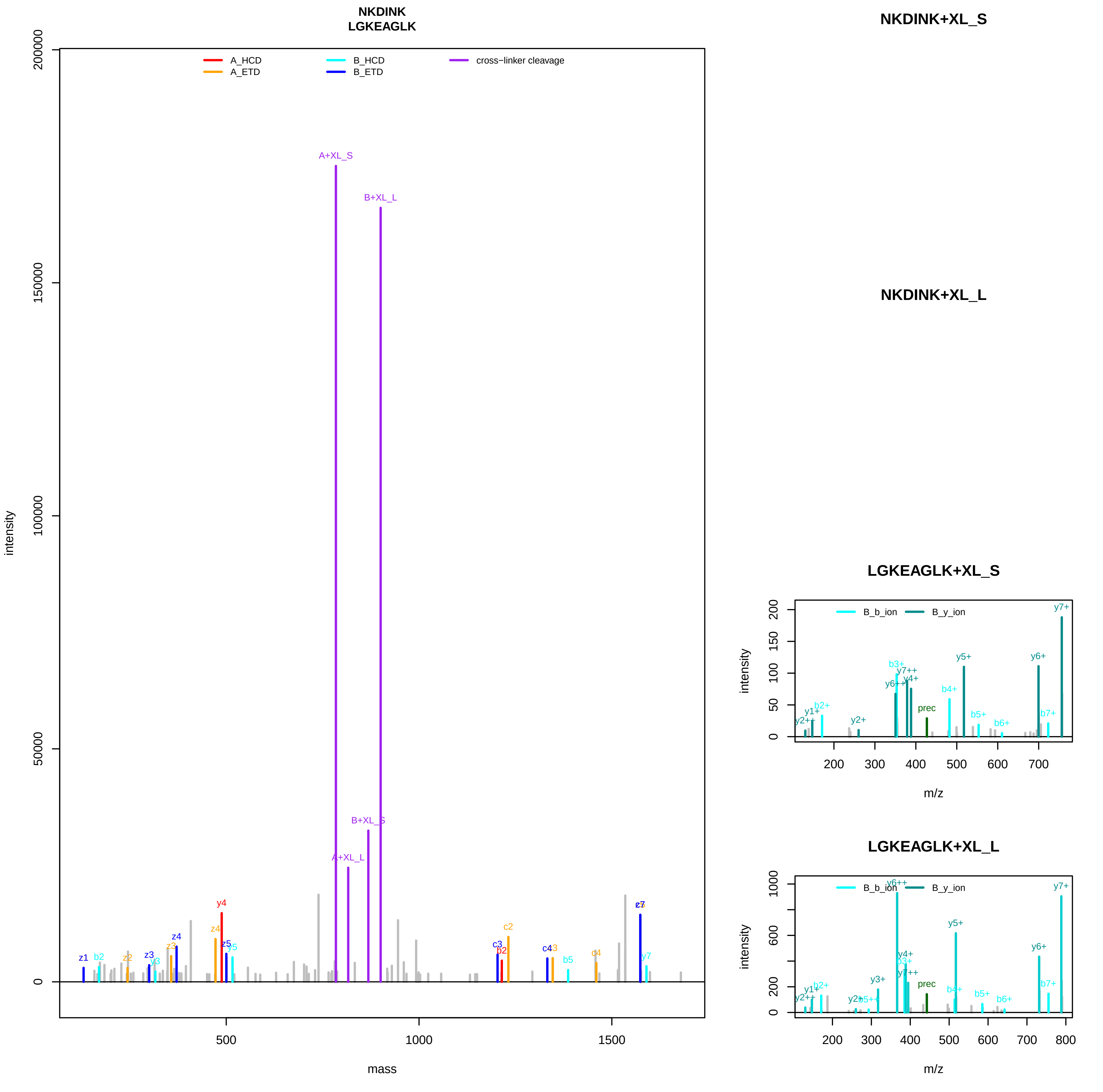
NKDINK
LGKEAGLK
A_HCD
A_ETD
B_HCD
B_ETD
cross−linker cleavage
A+XL_S
B+XL_L
B+XL_S
A+XL_L
z1
b2
z2
z3
y3
z3
z4
z4
y4
z5
y5
c3
b2
c2
c4
c3
b5
c4
z7
y7
intensity
mass
0
50000
100000
150000
200000
500
1000
1500
NKDINK+XL_S
NKDINK+XL_L
LGKEAGLK+XL_S
B_b_ion
B_y_ion
y2++
y1+
b2+
y2+
y6++
b3+
y7++
y4+
prec
b4+
y5+
b5+
b6+
y6+
b7+
y7+
intensity
m/z
0
50
100
150
200
200
300
400
500
600
700
LGKEAGLK+XL_L
B_b_ion
B_y_ion
y6++
y2++
y1+
b2+
y2+
b5++
y3+
y4+
b3+
y7++
prec
b4+
y5+
b5+
b6+
y6+
b7+
y7+
intensity
m/z
0
200
600
1000
200
300
400
500
600
700
800
